# Socio-Emotional Competencies and School Performance in Adolescence: What Role for School Adjustment?

**DOI:** 10.3389/fpsyg.2021.640661

**Published:** 2021-09-07

**Authors:** Nathalie Mella, Pascal Pansu, Anatolia Batruch, Marco Bressan, Pascal Bressoux, Genavee Brown, Fabrizio Butera, Anthony Cherbonnier, Céline Darnon, Marie Demolliens, Anne-Laure De Place, Pascal Huguet, Eric Jamet, Ruben Martinez, Vincent Mazenod, Estelle Michinov, Nicolas Michinov, Celine Poletti, Isabelle Régner, Mathilde Riant, Anais Robert, Ocyna Rudmann, Camille Sanrey, Arnaud Stanczak, Emilio Paolo Visintin, Eva Vives, Olivier Desrichard

**Affiliations:** ^1^Groupe de Recherche en Psychologie de la Santé (GREPS), University of Geneva, Geneva, Switzerland; ^2^Laboratoire de Recherche sur les Apprentissages en Contexte (LaRAC), University Grenoble Alpes, Grenoble, France; ^3^Laboratoire de Psychologie Sociale de l’Université de Lausanne (UnilaPS), University of Lausanne, Lausanne, Switzerland; ^4^Laboratoire de Psychologie Cognitive (LPC), UMR7290, Aix-Marseille University, Marseille, France; ^5^Laboratoire de Psychologie: Cognition, Comportement, Communication (LP3C), EA 1285, University of Rennes 1, Rennes, France; ^6^Laboratoire de Psychologie Sociale et Cognitive (LAPSCO), UMR-6024, University Clermont Auvergne, Clermont-Ferrand, France; ^7^Laboratoire d’Informatique de Modélisation et d’Optimisation des Systémes (LIMOS), UMR-6158, University Clermont Auvergne, Clermont–Ferrand, France

**Keywords:** socio-emotional competence, school adjustment, school performance, network analysis, self-regulation

## Abstract

There is growing evidence in the literature of positive relationships between socio-emotional competencies and school performance. Several hypotheses have been used to explain how these variables may be related to school performance. In this paper, we explored the role of various school adjustment variables in the relationship between interpersonal socio-emotional competencies and school grades, using a weighted network approach. This network approach allowed us to analyze the structure of interrelations between each variable, pointing to both central and mediatory school and socio-emotional variables within the network. Self-reported data from around 3,400 French vocational high school students were examined. This data included a set of interpersonal socio-emotional competencies (cognitive and affective empathy, socio-emotional behaviors and collective orientation), school adjustment measures (adaptation to the institution, school anxiety, self-regulation at school, and self-perceived competence at school) as well as grades in mathematics and French language. The results showed that self-regulation at school weighted the most strongly on the whole network, and was the most important mediatory pathway. More specifically, self-regulation mediated the relationships between interpersonal socio-emotional competencies and school grades.

## Introduction

Interest in socio-emotional competencies in education has grown considerably over the last few decades (Oberle et al., 2016) and there is now a large amount of research showing their positive and adaptive role at school (Durlak et al., 2011; Domitrovich et al., 2017).

A number of studies have shown that better socio-emotional competencies are associated with better school grades (Durlak et al., 2011; Taylor et al., 2017; MacCann et al., 2020).

Several hypotheses attempting to explain the positive relationships between socio-emotional competencies and school achievement have been suggested in the literature, but this issue is not clearly understood. Yet, while educational decision-makers in most western countries are beginning to consider the importance of socio-emotional competencies of pupils, a better understanding of the relationships between these competencies and school achievement may help to build strategies favoring their co-development. One hypothesis that comes from intervention studies assumes that better socio-emotional competencies would allow the pupils to better adapt to school and place them in conditions that are conducive to learning (Corcoran et al., 2018). School adjustment is believed to play a key role in the relationship between socio-emotional competencies and academic achievement. In this paper, we explored the role of several variables of school adjustment in the relationships between socio-emotional competencies and grades in French vocational high school students, using a weighted network approach. This approach is particularly interesting as it allowed us to visualize the structure and strength of interrelations between variables, and to identify direct and indirect mediatory pathways (Epskamp et al., 2012, 2018).

### Socio-Emotional Competencies at School

Socio-emotional competencies at school have been mainly explored in the framework of social and emotional learning (SEL), which organizes social and emotional competencies around five types of competence: self and social awareness, self-management, relationship skills and responsible decision-making (Greenberg et al., 2003; Jones and Doolittle, 2017). Programs targeting these competencies have been associated with positive development of a set of school adjustment variables, including decreased anxiety, better adaptation to the institution, more positive attitudes toward oneself, others and school, enhanced relationship quality, fewer disruptive behaviors, and improved self-regulation (McClelland et al., 2007; Durlak et al., 2011; Mega et al., 2014; Taylor et al., 2017). In addition, there is increasing evidence that these programs have a positive effect on school grades (Durlak et al., 2011; Taylor et al., 2017). A deeper exploration of the relationships between socio-emotional competencies and school grades highlighted the specific role of emotion understanding and emotion management (MacCann et al., 2020). These two competencies play a key role in interpersonal processes, which according to MacCann et al. (2020) are one of the main drivers of the relationships between socio-emotional competencies and school performance. In the present study, we focused on three interpersonal socio-emotional competencies: socio-emotional behaviors, empathy, and collective orientation.

Socio-emotional behaviors are at the root of adaptive social interactions. They encompass knowledge and respect of social rules, listening to and accepting others’ opinions, controlling negative emotions, and demonstrating positive behaviors in social situations, such as resolving conflicts or giving a positive image of friends. These overlap with the social awareness and relationship skills which are SEL concepts. Appropriate socio-emotional behaviors at school are required to develop good quality relationships with friends and teachers, and to comply with the school rules and systems. As mentioned above, programs designed to foster students’ social awareness and relationship skills, among the five SEL competencies, have shown to positively influence school achievement and that these effects last over several years (Durlak et al., 2011; Taylor et al., 2017). Socio-emotional behaviors have been related to school performance in various cultures (Chen et al., 2004).

Empathy is defined as the ability to understand and share others’ emotions. It comprises both cognitive and affective components. Cognitive empathy refers to the ability to adopt the perspective of others, also termed perspective-taking, and is a key process to understand and predict the intentions and emotions of others. It enables effective communication and interaction with others (Decety, 2005; Singer and Lamm, 2009). Affective empathy reflects the ability to share others’ emotions and to respond appropriately to others’ distress. This ability is believed to be at the root of pro-social behaviors, driving protection and help behaviors (Niezink et al., 2012). Both components of empathy are essential for social interactions, and have been put forward in the social awareness competence of SEL (Jones and Doolittle, 2017). At school, empathy is a foundation for healthy relationships with teachers and peers thus favoring a good adaptation to the institution. It may also contribute to positive learning experiences in collaborative settings (López-Mondéjar and Pastor, 2017).

Collective orientation refers to a team-oriented mindset that facilitates coordination and communication in group work (Driskell et al., 2010). It has been shown to predict performance on various tasks, including decision-making, negotiation and execution tasks (Driskell et al., 2010; Hagemann, 2017). This mindset is essential for successful collaborative learning. Research has shown that collaborative learning is effective if students have enough social skills to engage and interact in group work (Buchs and Butera, 2015). Collective orientation, as an index of a positive attitude toward group work, has been repeatedly shown to have benefits for social and cognitive outcomes, including better school performance (Johnson and Johnson, 2009; Stump et al., 2011).

### The Mediating Role of School Adjustment

Conceptual models of socio-emotional learning assume that socio-emotional competencies provide a foundation for better school adjustment, more positive attitudes toward school and one’s self, a more supportive learning environment and less school-related anxiety, which in turn lead to enhanced academic skills (Corcoran et al., 2018). This study focuses on a set of school adjustment variables, including adaptation to the institution, self-perceived school competence, school anxiety and self-regulation at school.

Adaptation to the institution refers to students’ abilities and their satisfaction with personal, social and emotional aspects of their integration into the school (Feldt et al., 2011; Credé and Niehorster, 2012). A good adaptation to the institution has been related to positive attitudes toward it and higher self-perceived competence (Martin et al., 1999), as well as to better socio-emotional competencies, such as emotion management abilities in university students (Nightingale et al., 2013). Meta-analytic results indicate that adaptation to the institution is also predictive of school performance (Credé and Niehorster, 2012).

Self-perceived school competence is also an important index of school adjustment. It has been associated with social adjustment, such as peer acceptance, and with socio-emotional competencies which are the ability to manage ones’ own emotion and to utilize emotions in social contexts (Harter and Leahy, 2001; Cicei et al., 2012). In addition, it has been found that self-perceived school competence is strongly related to academic performance (Chen et al., 2004; Valentine et al., 2004; Urdan and Schoenfelder, 2006; Chiu and Klassen, 2010; Huang, 2011). Some research has shown that self-perceived school competence and academic performance contribute to each other in a reciprocal manner (Muijs, 1997). In other words, students’ confidence in their abilities to achieve success influences and is affected by school performance. Some authors proposed that this reciprocal interaction is driven by behaviors that promote learning goals, such as self-regulation (Schunk and Zimmerman, 2012).

Self-regulation, or self-regulated learning, refers to “learning that results from students’ self-generated thoughts and behaviors that are systematically oriented toward the attainment of their learning goals” and a major factor of school adjustment (Schunk and Zimmerman, 2012, p59). Numerous studies have shown that students who adopt self-regulated strategies perform better at school (Weinstein and Acee, 2013). Self-regulation is an effortful process that implies executive control in the social, emotional and cognitive spheres, and individual differences in self-regulatory skills have been related to different empathy levels (Blair and Diamond, 2008; Eisenberg, 2010; Eisenberg et al., 2010). Although not all domains of self-regulation are related to learning (Sitzmann and Ely, 2011), we may hypothesize that self-regulatory processes needed for self-management in social and school contexts share common bases, and that part of the relationships between socio-emotional competencies and school achievement is explained by this common mechanism. Research has shown that self-regulated learning mediates the effects of emotions on academic achievement in undergraduate students (Mega et al., 2014). It is therefore likely that self-regulation at school will be linked to socio-emotional competencies in the network of positive competencies which lead to good school adaptation.

Lastly, school anxiety is a substantial indicator of school non-adjustment, reflecting a lack of emotional wellbeing. Different forms of anxiety including test anxiety, separation anxiety or social anxiety, may occur at school and give rise to general symptoms of anxiety. These symptoms are characterized by negative thoughts (e.g., hopelessness, worry, and fear), physiological reactions (e.g., sweating, upset stomach, and tremors) and/or inappropriate behaviors such as avoidance or procrastination, which may affect the socio-emotional life at school, academic strategies and performance (McDonald, 2001; Wren and Benson, 2004; Chapell et al., 2005; Pascoe et al., 2020). In a recent review, Pascoe et al. (2020) reported consistent adverse effects of school anxiety on academic performance, socio-emotional life at school, and self-regulated learning. Test anxiety has also been shown to be negatively related to self-perceived school competence (Raufelder and Ringeisen, 2016).

Overall, the literature supports reciprocal interrelations between interpersonal socio-emotional competencies, school adjustment and academic performance. However, most studies used methods that do not facilitate the visualization of the structure or a study of the strength of these interrelations. A weighted network approach overcomes these limitations.

### The Network Approach in Psychology

The network approach is being increasingly used to visualize and analyze psychological data. This approach considers abilities, personality traits, symptoms and behaviors as directly affecting each other. For example, the network approach has been used in the field of psychopathology (Borsboom and Cramer, 2013; Fried et al., 2017), personality (Costantini et al., 2019) and health psychology (Nudelman et al., 2019) to give a descriptive view of the patterns of co-occurrence of symptoms and health behaviors. Unlike traditional analyses exploring multiple associations, this approach allows the researcher to identify how central constructs are linked in a given network. Centrality is of fundamental interest as it reveals the degree of connectedness with the entire network: a node with a high degree of centrality will act as a hub connecting the whole network and will therefore have an influence on the entire network (Bringmann et al., 2019). Some indexes of centrality also identify nodes that act as mediators between different variables or between a subnetwork of variables, by taking into consideration the frequency with which a given node lies in the shortest path between two other nodes. This approach is therefore useful to explore (1) weighted interrelations between socio-emotional competencies, school adjustment and school grades and (2) the role of various potential mediators between socio-emotional competencies and school grades. Furthermore, the network approach allows the researcher to better understand the weighted role of each variable within the network, thus avoiding the aggregation of data that often masks a finer granularity of how each competence, characteristic or performance interrelate.

### Objectives

In this paper, we used a network approach to gain a deeper understanding of the nature and structure of the interrelationships between interpersonal socio-emotional competencies school adjustment and school performance. We analyzed data from a large study of French vocational high school students (ProFan). Vocational high schools are part of the French education system enabling pupils to learn a trade by gradually moving from the school environment to the world of work often through dual training courses alternating between the workplace and school. Vocational high school students constitute a heterogeneous population mainly from low to average income socio-economic backgrounds, and whose socio-emotional and school functioning is largely under-explored. In this study, we assessed: (1) three interpersonal socio-emotional competencies–socio-emotional behaviors, empathy and collective orientation; (2) four school adjustment measures–adaptation to the institution, self-perceived school competence, self-regulation at school, and school anxiety; and (3) grades in French language and mathematics. We expected all the variables to be directly or indirectly interrelated, as they are all linked to school adjustment.

## Materials and Methods

### Participants

The sample is drawn from the ProFan project, a large research project launched in 2017 by the French Ministry of Education, which tested the impact of collaborative learning on several major outcomes, such as socio-emotional functioning, academic performance, or school adaptation. Two cohorts of students from 109 French vocational high schools participated in this longitudinal research project. Their vocational studies were business, health services, and electricity. The high schools included in the study were chosen by the French Ministry of Education, on a voluntary basis. The classes were selected randomly in these schools, and the study was part of the curriculum, so that the students were obliged to participate. This analysis focuses on data from the initial baseline of the second cohort, which was collected in October-December 2018 from 4342 students (57% females, mean age 16.41 years).

### Procedure

The data were collected during school hours in the schools’ computer rooms. The students had to fill out an online questionnaire containing 251 items. This questionnaire included items relating to socio-emotional competencies, school adjustment, and academic performance. Other items related to beliefs and attitudes concerning school or learning, and items relating to students’ functioning in class were also part of the questionnaire, but were not used in this analysis (see Supplementary Appendix D). The questionnaire was presented online using a specially designed internet platform.

#### Measures of Socio-Emotional Competencies

##### Socio-emotional behaviors

We used a scale developed by Lippman et al. (2014) to assess the extent to which the participant uses a set of positive skills necessary to get along well with others and function constructively in groups. These skills include expressing respect and appreciation for others, working efficiently with others, presenting ideas and listening to others’ ideas, regulating emotions, behaving according to social norms, and using conflict resolving skills. The scale had eight items (e.g., “I control my anger when I have a disagreement with a friend”), for which the participants had to indicate if the statement was “exactly like [them]” to “not at all like [them]” on a five point Likert scale (Cronbach alpha = 0.69).

##### Empathy

The Interpersonal Reactivity Index (IRI) (Gilet et al., 2013) was used to assess both cognitive and affective empathy: (1) cognitive empathy: the ability to adopt another person’s point of view (subscale: perspective-taking), (2) affective empathy: the tendency to experience compassion for others (subscale: empathic concern. Each subscale contained four items, for which the participants had to indicate whether they agreed with the statements (e.g., “Before criticizing somebody, I try to imagine how I would feel if I were in their place.”) on a 7 point Likert scale from “strongly agree” to “strongly disagree” (Cronbach alpha = 0.76 for empathic concern and 0.64 for perspective-taking).

##### Affiliation

An eight-item subscale of the Collective Orientation Scale was used to assess the ability and tendency to value working with others as opposed to working alone (Driskell et al., 2010). For each item, the participants had to indicate if they agreed with a statement (e.g., “I can usually perform better when I work on my own”) on a five point Likert scale from “totally agree” to “totally disagree” (Cronbach alpha = 0.76).

#### Measures of School Adjustment

##### Self-perceived competence at school

It was assessed using the French version of the five-item perceived school competence subscale (Bariaud, 2006) from the Self-Perception Profile for Adolescents (Harter, 2012). Each item presented a description of two groups of students with opposite characteristics. Participants were asked to select the group that best described them and assess how true that assertion was for them (“really true for me” or “sort of true for me”). Each item had a score from one to four, and the responses were averaged to obtain a subscale score. A high score indicated a high self-perceived competence at school. The internal consistency was moderate, but acceptable (Cronbach alpha = 0.67).

##### Self-regulation at school

Nine items adapted from the Motivated Strategies for Learning Questionnaire (Pintrich, 1991) were used to assess self-regulated behaviors at school, including the ability to concentrate and motivate oneself (e.g., “I am able to work on my own at home and at school”) or to work independently (e.g., “I prepare myself before an exam”). For each item, the participants had to answer using a seven point Likert scale from “totally disagree” to “totally agree” (Cronbach alpha = 0.86).

##### Adaptation to institution

We used the Student Adaptation to College Questionnaire (SACQ) (Dahmus et al., 1992) to asses general adaptation to the school environment. This questionnaire had sixteen items which assessed three dimensions: social adaptation (e.g., “If I feel depressed, my friends will help me to get better”), attachment to institution (e.g., “sometimes, I would like to give up everything”), emotional and personal adaptation (e.g., “I can’t get used to my life here”). A global score of general adaptation to the institution was computed from the 16 items using a seven point Likert scale ranging from “totally disagree” to “totally agree” (Cronbach alpha = 0.84).

##### School anxiety

We used a questionnaire adapted from the State-Trait Anxiety Inventory for Children (STAIc) (Turgeon and Chartrand, 2003), in order to assess school anxiety. The French version adapted by Pouille (2016) contains (e.g., “when I go to school, my heart beats fast”), for which participants had to indicate the frequency with which they experienced the symptoms on a four point Likert scale from “never” to “always” (Cronbach alpha = 0.85).

#### Measures of School Performance

The participants were asked to provide their grades in mathematics and French language obtained the year preceding the data collection. Self-reported grades have been shown to be reliable, especially for children from grades 9 to 11 (Sticca et al., 2017). Grades in the French education system are based on a 20-point scale. We collected self-reported grades on a five-point scale (1 = 0–4.9; 2 = 5–8.9; 3 = 9–12.9; 4 = 13–15.9; and 4 = 16–20) to avoid over or under reports.

#### Socio-Demographic Measures

The students’ socio-economic status (SES) was coded according to the parents’ occupational status from 1 (low SES) to 4 (high SES). The highest occupational status in the family was selected for the analysis. The majority had a low SES (40% = 1; 27% = 2; 25% = 3; and 8% = 4).

### Data Preparation

To remove invalid data, we computed a variability score for each scale and each participant, using an intraindividual standard deviation (iSD) coefficient. We chose to remove data from each participant showing no variation (iSD = 0) in scales using reversed items, which suggested that they had systematically responded with the same pattern of responses, independently of the meaning of the items. Around 20% of the data were thus removed, leading to a final sample of 3,385 participants aged 15–20 years (60% female, mean age 16.35 years).

### Analysis

#### Network Construction^1^

Partial correlation networks were computed using the R package qgraph (Epskamp et al., 2012; Epskamp and Fried, 2018). The full R code for these analyses can be found in the Supplementary Appendix B. We performed a lasso regularization of the networks using EBIC selection (hyperparameter λ = 0.5) to optimize the network (i.e., aiming to include as few false positives as possible, or not estimating connections that were not true in the network) (Epskamp et al., 2012). All variables were then input into the analysis. The edges were estimated to be above 0.10, meaning that the measures had substantial connections to each other (Cohen, 1988). In the networks, partial correlation coefficients were close to multiple regression coefficients (Epskamp and Fried, 2018). In addition, this model may display potential mediating variables (e.g., A → B → C would show that B mediated the relationships between A and C).

#### Centrality

Two centrality indexes were computed: Strength and Betweenness. Strength is the sum of weighted values of its connections with other nodes in the network. It provides information on the importance of the node in the network and therefore its potential influence over the entire network (Bringmann et al., 2019). Betweenness reflects the frequency with which a given node lies in the shortest path between two other nodes. It is an indicator of indirect connections and provides information on the mediatory paths between several nodes of the network. These centrality indexes were assessed using the R package *qgraph* (Epskamp et al., 2012). A central node (high strength) with high betweenness will rapidly affect the whole network and reciprocally (Epskamp et al., 2018).

## Results

**FIGURE 1:**
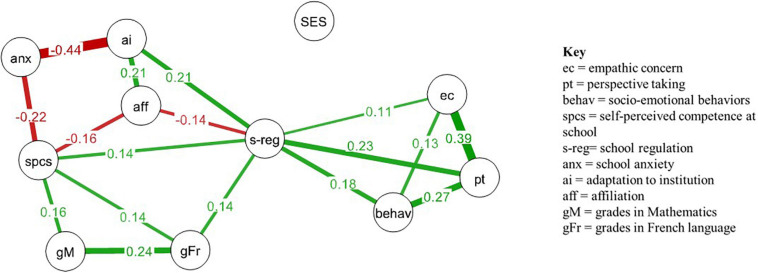
Weighted network of socio-emotional competencies, school adjustment and school grades (*r* ≥ 0.10 are displayed). Green lines represent positive correlations, red lines represent negative correlations. Thin lines represent weak connections and thick lines represent strong connections.

The network concerning socio-emotional competencies is displayed in Figure 1, and the centrality nodes of this network are shown in Table 1.

**TABLE 1:** Nodes centralities for each variable of the network.

Variables	Betweenness	Strength
**s-reg**	**19**	**1.155**
pt	8	0.886
ai	5	0.859
spcs	5	0.813
anx	2	0.656
ec	0	0.631
behav	0	0.590
gFr	4	0.520
aff	0	0.513
gM	0	0.404
SES	0	0.000

*cont = emotional contagion; ec = empathic concern; pt = perspective-taking; behav = Socio-emotional behaviors; psc = perceived socio-emotional competence; spcs = self-perceived competence at school; s-reg = school regulation; anx = school anxiety; ai = adaptation to institution; aff = affiliation; gM = grades in Mathematics; gFr = grades in French language; SES = socio-economic status. Nodes with the highest centrality values are in bold.*

A descriptive analysis of the global structure of the network shows that this network is dense, as most of the nodes are weakly to moderately interconnected. Two distinct parts emerge from visual inspection of the network: both affective and cognitive empathy and socio-emotional behaviors are strongly and positively interrelated, and separated from the rest of the network by self-regulation. As expected, grades in mathematics and French language show no direct relationships with socio-emotional competencies, but indirect connections through self-regulation.

Self-regulation shows the highest score of betweenness centrality (Table 1)–more than double the second betweenness centrality variable–indicating that this variable has the most indirect connections with other nodes in the network. In addition, self-regulation displays the highest strength centrality, suggesting that this variable is not only an important mediatory variable, but it also weighs heavily on other network variables. It shows direct connections with all socio-emotional competencies and with grades in French language, adaptation to institution and self-perceived competence at school. Conversely, it only shows an indirect connection with grades in math and school anxiety. In other words, it has both strong direct and indirect connections with the whole network.

Unexpectedly, collective orientation (affiliation) is not related to other socio-emotional competencies. It is negatively related to self-regulation and self-perceived school competence, but positively to adaptation to institution. In addition, it is negatively linked to school grades via indirect connections.

School grades are only positively related to self-perceived competence at school for French language and mathematics and self-regulation in French language. All the other network variables only show indirect connections with school grades.

As expected, school anxiety has negative connections with self-perceived competence at school. This is also the case for adaptation to the institution, which is the strongest connection in the entire network.

Lastly, parental SES was not related to any variable of the network, indicating that the structure of the interrelations between interpersonal socio-emotional competencies, school adjustment and grades is similar whichever the SES.

## Discussion

Considering socio-emotional functioning, school adjustment and school performance from a functional network perspective allowed us to explore the strength and structure of their interrelations, and to investigate variables that are central within the network and their mediatory paths. Our results showed that (1) socio-emotional competencies, school adjustment and school performance are highly interconnected, (2) self-regulation is a central variable in these functional network, both in terms of strength and as mediatory variables between socio-emotional competencies and school performance.

### A Highly Interconnected Network

As expected, variables assessing socio-emotional competencies, school adjustment and school grades were highly interconnected, and form a network of positive functioning at school, adaptive variables being positively related to other adaptive variables, and school anxiety showing negative relationships with adaptation and self-perceived competence at school.

### The Central Role of Self-Regulation at School

Academic self-regulation is the most central variable of the network, both in terms of strength and of betweenness. It has connections with virtually all the network variables and mediates the relationships between socio-emotional functioning and other school adjustment variables and school grades. The relationship between self-regulatory abilities and academic performance has been extensively studied, school self-regulation being one of the strongest predictors of school performance (Zimmerman and Schunk, 2001; Komarraju and Nadler, 2013; Gestsdottir et al., 2014). The relationship between self-regulation at school and socio-emotional competencies has been less explored. Our results showed that self-regulated abilities were related to cognitive empathy, affective empathy and to socio-emotional behaviors. Cognitive empathy, referring to perspective-taking abilities, and the regulation of social behaviors both strongly rely on the cognitive aspects of socio-emotional processing and one may hypothesize that the relationships between school self-regulation and socio-emotional competencies depend on the ability to deploy effective strategies. This supports the idea that the same overarching socio-emotional cognitive factors are important for the regulation of academic work and socio-emotional adaptation (Patrick, 1997). It echoes the recent hypothesis that the regulation of academic emotions is an important mechanism by which socio-emotional competencies influence school grades (MacCann et al., 2020). Furthermore, affective empathy has a weaker connection with self-regulation (*r* = 0.11) than cognitive empathy (*r* = 0.23).

Self-regulation was also positively related to adaptation to institution and to self-perceived competence at school, indicating that students with good self-regulated learning strategies have a more positive view of their scholastic abilities and feel a stronger sense of belonging in their school, and *vice versa*. These interrelations may be explained by a dynamic interaction between positive representations of the self, of others and the school, and the use of effective learning strategies, which could be driven by a general feeling of self-management control (Isen, 2000). Earlier studies have shown that self-perceived competence at school, social behaviors and school grades are consistently related in adolescents from different cultures (Chen et al., 2004). Our results point to the essential role of self-regulation in these relationships.

As our study was conducted among high school adolescents, there may be a different pattern with younger children. Late adolescence is indeed a critical period during which self-regulation is directly related to mental and physical health, social relationships and psychological adjustment (Park et al., 2012; Farley and Kim-Spoon, 2014). Research has shown that self-regulatory abilities increase steadily from pre-adolescence to young adulthood, and that adolescence is a key period for a reorganization and development of regulatory systems (Steinberg, 2005; Steinberg et al., 2018). Although these abilities have a significant impact on diverse outcomes in early childhood, such as better school performance, it is likely that self-regulation gradually becomes a more central process in adolescents’ school adaptation. Future studies could explore this subject with younger children.

### Variables Connected to School Grades

Grades in mathematics and French language were not central network variables, and showed very low indexes of betweenness and strength centrality. As stated before, school grades were not directly related to socio-emotional competencies, but indirectly *via* self-regulation. Grades in math were only related to self-perceived competence at school, while grades in French language were related to self-perceived competence at school as well as self-regulation. self-perceived competence at school was therefore the most consistent factor associated with school performance. Numerous studies have pointed to a strong and persistent relationship between self-perception of academic abilities and academic performance (Purkey, 1970; Huang, 2011).

### Maladjustment Variables in the Network

As mentioned above, collective orientation showed unexpected connections with other network variables. It was negatively related to both self-perceived competence at school and school self-regulation, and therefore indirectly negatively related to school performance. This observation suggests that group work may be more beneficial to vulnerable students, i.e., those showing lower levels of school adjustment. Alternatively, this negative relationship might be the reflection of poor adjustment of learning and evaluative systems to the group work itself. However, collective orientation showed a positive relationship with adaptation to institution, which may be explained by a common positive disposition toward social environments. Thus, affiliation tendencies could enable students to get along with other classmates in group work situations while also reinforcing social adaptation to the school environment. Conversely, school anxiety was related to lower levels of adaptation to institution and to self-perceived competence at school. Research exploring relationships between internalized problems, school grades and self-perception has shown that self-perception mediated the pervasive effect of school grades on internalized problems (Metsäpelto et al., 2020). Other studies have shown that test anxiety is more strongly related to self-perceived competence at school than to actual school performance (Meece et al., 1990). Consistent with these findings, our results suggest that emotionally vulnerable adolescents have a lower self-perception of their academic competence and/or that a low perception of academic competence generates school anxiety, and that self-perception of competence mediates the relationships between anxiety and grades.

### Limitations

The first limitation of our study is the restricted number of socio-emotional competencies taken into account in this analysis. Additional emotional and social skills, such as emotion regulation and emotion recognition, would have provided more information about the architecture of socio-emotional functioning and how it relates to school adjustment. As the network approach depends on the variables used in the analysis, extending our results to a wider socio-emotional network would be necessary to consolidate our findings. This limitation also applies to the potential mediatory variables in the network. The literature highlights several school environment factors and individual characteristics that can also influence school achievement. For example, McCormick et al. (2015) have shown that the positive impact of a socio-emotional learning program on reading abilities was mediated by improved classroom organization. Better socio-emotional competencies might indeed facilitate class climate and organization. Other authors have put forward factors linked to personality and intelligence (e.g., MacCann et al., 2020). As our analysis focused on the benefits of a network approach, future research could extend its application to a broader analysis of socio-emotional competencies and school adjustment. Another limitation is the use of self-reported grades, which according to some studies, could be less reliable than objective grades (Kuncel et al., 2005). Due to the strict data privacy rules enforced within the French education system, we were unable to access students’ grades ourselves.

## Conclusion and Perspectives

The use of a network approach helped us to better understand the architecture relating to socio-emotional competencies and school adjustment, giving a new perspective of their relationships with each other. Analyzing the structure of this network allowed us to make two major advances: (1) adopting an exploratory approach to determine the mediatory variables relating socio-emotional competencies to school performance, and (2) identifying variables that are the most central in the positive network of school adaptation. Our results reveal that, at adolescence, good self-regulation abilities at school are central to both social and affective aspects of adaptation to school and school achievement. This result may have significant implications for education. One application of our results could be the implementation of teaching programs designed to develop students’ self-regulation abilities, i.e., organizational, self-management skills, to strengthen school adaptation. For example, some aspects of professional time management training courses could be used in these teaching programs. Our results also suggested that more cognitive socio-emotional competencies, such as perspective taking skills, have a stronger weight on the network of school adaptation than other socio-emotional variables. Keeping in mind the limitation due to the restricted number of socio-emotional competencies in our study, a potential application would be to include perspective taking as a major component of socio-emotional programs at school.

## Data Availability Statement

The raw data supporting the conclusions of this article will be made available by the authors, without undue reservation.

## Ethics Statement

Ethical review and approval was not required for the study on human participants in accordance with the local legislation and institutional requirements. Written informed consent from the participants’ legal guardian/next of kin was not required to participate in this study in accordance with the national legislation and the institutional requirements.

## Author Contributions

NaM analyzed the data. NaM and PP wrote the manuscript. OD proofread the different versions. All authors contributed to the data collection, designed the experiment and approved the submitted version.

## Conflict of Interest

The authors declare that the research was conducted in the absence of any commercial or financial relationships that could be construed as a potential conflict of interest.

## Publisher’s Note

All claims expressed in this article are solely those of the authors and do not necessarily represent those of their affiliated organizations, or those of the publisher, the editors and the reviewers. Any product that may be evaluated in this article, or claim that may be made by its manufacturer, is not guaranteed or endorsed by the publisher.

## Footnotes

1 Although we did not have any hypothesis concerning any gender effect in the structure and strength of interrelations in the network. We ran two exploratory analyses to compute different networks for girls and boys. It is indeed not possible to include both binary and non-binary variables in the network to control for or explore the effect of gender (Van Borkulo et al., 2014). The networks can be viewed in the Supplementary Appendix B. The networks show similar structures and central variables for boys and girls.

## Supplementary Material

Supplementary Appendix A Correlations between all variables of the network.

Supplementary Appendix B Weighted networks for boys and girls separately.

Supplementary Appendix C R code for the network analysis.

Supplementary Appendix D List of other indicators assessed in the PROFAN study.
