# Supplementary material for: Socio-Emotional Competencies and School Performance in Adolescence: What Role for School Adjustment?
Source: Front Psychol. 2021 Sep 7;12:640661. doi: 10.3389/fpsyg.2021.640661 (PMC8452941; doi:10.3389/fpsyg.2021.640661)
Supplement: Supplementary Appendix A — Correlations between all variables of the network. [file Data_Sheet_1.PDF]

Supplementary Materials

Appendix A

Table 1: Correlations between all variables of the network

	**gFR**	**gM**	**pschc**	**anx**	**aff**	**ai**	**pt**	**behav**	**ec**
gM	.33*								
pschc	.26*	.27*							
anx	-.03	-.06*	-.32*						
aff	-.11*	-.07*	-.17*	-.08*					
ai	.12*	.11*	.26*	-.49*	.20*				
pt	.11*	.07*	-.02	-10*	.09*	.12*			
behav	.17*	.12*	.10*	.00	.08*	.20*	.48*		
ec	.12*	.05	-.03	.11*	.07*	.11*	.55*	.39*	
s-reg	.29*	.23*	.26*	-.07*	-.10*	.30*	.43*	.41*	.35

Note: ec = empathic concern; pt = perspective taking; behav = Socio-emotional behaviors; pschc = perceived self-competence at school; s-reg= school regulation; anx = school anxiety; ai = adaptation to institution; aff = affiliation; gM = grades in Mathematics; gFr = grades in French language. * = p < .001.

Appendix B: Weighted networks in boys and girls

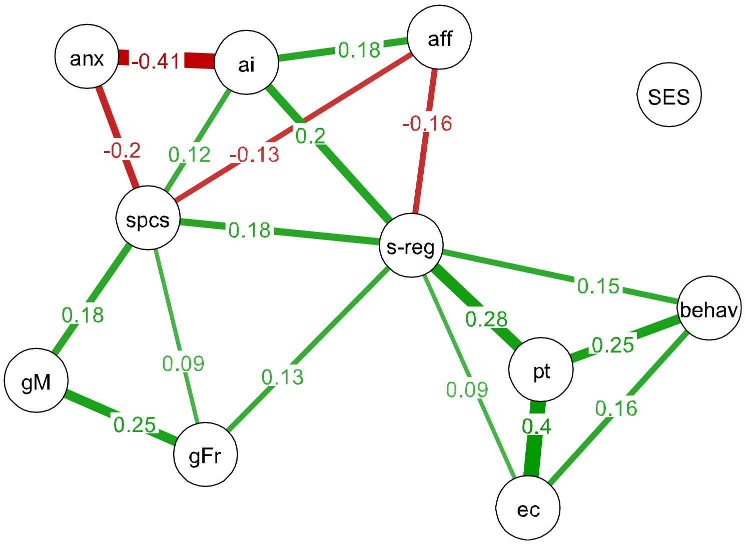

Key
ec = empathic concern
pt = perspective taking
behav = socio-emotional behaviors
spcs = self-perceived competence at school
s-reg= school regulation
anx = school anxiety
ai = adaptation to institution
aff = affiliation
gM = grades in Mathematics
gFr = grades in French language

Figure 1: Weighted network of socio-emotional competences, school adjustment and school grades in boys. Green lines represent positive correlations, red lines represent negative correlations. Thin lines represent weak connections and thick lines represent strong connections.

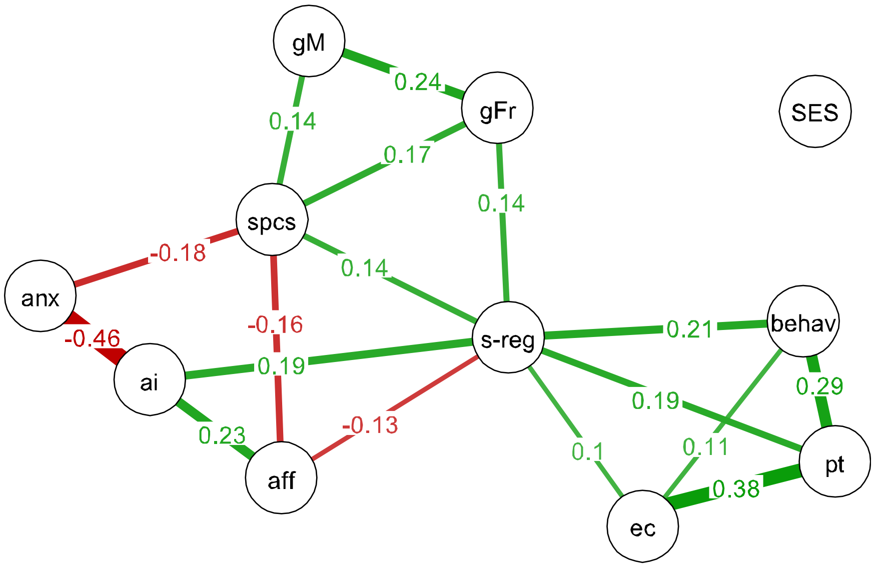

Key
ec = empathic concern
pt = perspective taking
behav = socio-emotional behaviors
spcs = self-perceived competence at school
s-reg= school regulation
anx = school anxiety
ai = adaptation to institution
aff = affiliation
gM = grades in Mathematics
gFr = grades in French language

Figure 2: Weighted network of socio-emotional competences, school adjustment and school grades in girls. Green lines represent positive correlations, red lines represent negative correlations. Thin lines represent weak connections and thick lines represent strong

Appendix C: R code for the network analysis

```
# Packages
"qgraph";"kableExtra"

# Naming variables
names (d)
vars <- c( "smoyFR", "smoyMath", "sec", "spd",   "spt",  "sscts",   "sautoeff",   "sest_scol", "sanx",  "saff","scaq_g", "sautoreg")
names (d) [which (names (d) %in% vars)] <- c("moyFR", "moyMath", "ec", "pd", "pt", "scts", "autoeff", "est_scol","anx", "aff","caq_g", "autoreg")
names (d)
vars <- c("moyFR", "moyMath", "ec", "pd", "pt",  "scts", "autoeff","est_scol", "anx", "aff","caq_g", "autoreg")

# correlation between variables
kable (cor (d[,vars], use = "pairwise"), digits = 3, caption = "Les corrélations entre les variables").
r1 <- qgraph (cor (d[,vars], use = "pairwise"), graph = "cor", edge.labels = T, layout = "circle")

# partial correlations between variables
mcor <- cor_auto(d[,vars], detectOrdinal = FALSE)
r2 <- qgraph (mcor, graph = "pcor", edge.labels = T, edge.label.position = 0.4, colFactor = 0.8, layout = "spring")

# regularized network using glasso (hyperparameter = 0.5)
R3 <- qgraph (mcor, weighted=TRUE, graph = "glasso", tuning = 0.5, threshold = .099, sampleSize = 3456, edge.label.cex = 1.2, edge.labels = T, edge.label.position = 0.3, palette = "grey", colFactor = 0.2, layout = "spring")

# Centrality indexes
centrality_auto(r3)
```

Appendix D: List of other indicators assessed in the PROFAN study.

- Perceived global self-worth, perceived job competence (Harter, 2012)
- Perceived competence in Mathematics and French language (adapted from Harter, 1985)
- Perceived parental dissatisfaction and perceived parental pressure.
- Perceived support in the classroom (Williams & Deci, 1996)
- Motivation in Mathematics and French language (Ryan & Connell, 1989)
- Motivation for cognitive activities (Desrichard et al., in prep)
- Self-efficacy, attitude and subjective norms towards socio-emotional competencies (Desrichard et al., in prep)
- Implicit theories of intelligence (Da Fonseca et al., 2004)
- Beliefs in school meritocracy (Wiederkehr et al., 2015)
- Social comparison tendencies (Gibbon & Buunk, 1999)
- Creative cognition (Rogaten & Moneta, 2015)
- Achievement goals at school (Darnon & Butera, 2005)
- Sensitivity to error (Seidah et al., 2002)
- Impostor syndrome at school (Bouffard et al., 2011)
- Verbal competencies (Bonnardel, 1940)
